# Overview of Current Practices in the Methamphetamine Testing and Decontamination Industry: An Australian Case Study

**DOI:** 10.3390/ijerph18178917

**Published:** 2021-08-25

**Authors:** Emma J. Kuhn, G. Stewart Walker, Harriet Whiley, Jackie Wright, Kirstin E. Ross

**Affiliations:** 1 College of Science and Engineering, Flinders University, Adelaide, SA 5001, Australia; stewart.walker@flinders.edu.au (G.S.W.); harriet.whiley@flinders.edu.au (H.W.); jackie.wright@flinders.edu.au (J.W.); kirstin.ross@flinders.edu.au (K.E.R.); 2 Environmental Risk Sciences, Sydney, NSW 2118, Australia

**Keywords:** methamphetamine contamination, third-hand exposure, testing, remediation, industry practices, regulation, guidelines, public health

## Abstract

To better protect public health from third-hand exposure to methamphetamine, it is important to understand the techniques and current practices used within the methamphetamine testing and decontamination industry in Australia. A survey was conducted focusing on business owners that advertised testing and/or remediation services online. They were also invited to participate in a follow-up phone interview upon completion. The survey demonstrated that testing and decontamination methods were highly varied, which was expected for an industry with no regulation. Most companies offered methamphetamine testing and remediation which could be a conflict of interest. Participants also shared personal experiences, including the conduct of other industry members, demonstrating both poor practice and/or the competitive nature of the business. Participating business owners were following Australian guidelines to the best of their ability, and many are advocates for regulation to be implemented within the industry. This would address the inconsistencies between companies and establish trust for industry members and the public. It would also provide significant public health protection, which is currently lacking. A more consistent approach to the testing and remediation of methamphetamine contamination, aided by regulation, would address the significant risk to public health caused by third-hand exposure to methamphetamine.

## 1. Introduction

Methamphetamine contamination of properties is an emerging public health issue. Methamphetamine is a highly addictive amphetamine-type stimulant that is commonly used and manufactured within residential properties [1,2]. This illegal drug can be inhaled via smoke, ingested, or injected [3], and has different forms, including a crystalline structure, a waxy base, and a whiteish powder [4]. During the illegal manufacturing of methamphetamine or through personal usage (smoking), methamphetamine vapours are released into the air and absorbed by materials such as walls and furniture [5]. It has been shown that methamphetamine residues can remain present for over five years and contaminate new furniture introduced to the property [5]. These residues can significantly impact resident’s behaviour, cognitive function, and general health [6]. Through chronic exposure, health effects include eye and skin issues, headaches, respiratory effects, and sleep disturbances [6,7,8]. The environmental contamination exposure time in adults and children is highly dependent on the individual, the activities they undertake, and the time spent within the property [6]. Due to the broad nature of the symptoms associated with third-hand exposure to methamphetamine, the lowest observed adverse effect level is difficult to determine with the existing data. Third-hand exposure to methamphetamine remains an under-researched area of environmental health.

The methamphetamine remediation industry is comprised of occupational hygienists and other testing companies (for testing and validation) and remediation businesses. Many companies within the methamphetamine remediation (MR) industry have MR as an added service to existing cleaning business models. Subsequently, equipment, processes, and chemicals from domestic or commercial cleaning have also been adapted to MR. In Australia, when a property is suspected of methamphetamine contamination, the tenants or owners seek advice from a testing and/or remediation company that will conduct an assessment on the level of contamination. Prior to any cleaning, a Remediation Action Plan will be produced to define the extent of remediation required. The property is then remediated, and to ensure it has been adequately cleaned, a validation test will be conducted to assess any residual contamination. In line with Australian guidelines, qualified occupational hygienists conduct the validation tests and determine whether the property has been decontaminated.

In Australia, advice and recommendations are determined through the Australian Clandestine Drug Remediation Guidelines (AG) and, more recently, the Australian Voluntary Code of Practice (VCOP); however, there is currently no legislation or regulation for MR [5,9]. As a result, this industry has no mandatory training, nor standardised techniques that are specific to MR. In Australia, the contamination investigation level is for surfaces only, and is 0.5 µg per 100 cm^2^ [9]; New Zealand standard’s limit is 1.5 µg per 100 cm^2^ [10]. In the United States, these limits vary depending on the state, from 0.05 µg per 100 cm^2^ in Arkansas to 4.0 µg per 100 cm^2^ in Colorado [11,12,13]. These risk-based limits have been adopted as the remediation criteria based on what is considered to be an acceptable level. These standards offer guidance; however, they are not enforceable in Australia nor New Zealand as they have not been incorporated into legislation. The regulation of remediating methamphetamine-contaminated properties is critical to protect human health from this emerging public health risk.

The aim of this study was to determine the methods, treatments and protocols currently used within the MR industry, using Australia as a case study. A secondary aim was to gain insight into the general practices of cleaning companies offering an MR service to the public. To conduct this, companies advertising MR services were contacted and asked about their activities through an online survey, followed by one-on-one interviews, where possible.

## 2. Materials and Methods

### 2.1. Identifying Potential Participants

Companies advertising an MR or testing service in Australia were identified using the search engine Google^®^ (Google, Menlo Park, CA, USA) And the following key search terms were used: methamphetamine OR ‘clandestine lab’ OR ‘meth lab’ AND remediation AND contaminated OR contamination AND Australia AND company OR business. A list was generated with company contact details. Owners with multiple business names were only listed once.

### 2.2. Survey Questions

The research methodology and survey were approved by the Flinders University Social and Behavioural Research Ethics Committee (SBREC) in South Australia (Project number 8634). The survey was conducted online (Qualtrics^®^ software (Qualtrics, UT, USA)) and included single-answer multiple-choice questions, multiple-answer multiple-choice questions and free-text questions (Supplementary S1). The varied question styles were designed to permit the participant to add as much or as little detail for questions based on their experience or practice. Using only multiple choice questions would have hindered openly honest answers; however, they did provide structure and time efficiency for the remainder of the survey [14].

### 2.3. Contacting Companies

To initiate contact with business owners, an email was sent with an introduction, information attachment, and the link to the survey. The Qualtrics^®^ link remained open for two months to allow participants to complete the survey without time pressure.

### 2.4. Phone Interviews

Upon completion of the online survey, participants were given an option to be contacted via phone to provide further details regarding their survey answers. Those participants that indicated that they were willing to participate in a one-on-one interview were contacted by phone for approximately ten minutes to discuss their survey responses. These interviews were transcribed into Microsoft Word^®^ (Microsoft, Redmond, WA, USA) for qualitative data analysis.

## 3. Results

### 3.1. Participants

The online search identified 100 companies involved in methamphetamine remediation in Australia. This included 64 individual business websites and 36 franchise contacts. Of the 64 websites, 14 shared ownership with another business, and of the 36 franchises, 10 franchises owned other locations. There was also one business that no longer existed. Thus, there were 75 individual owners that could be contacted to participate in the study. Of the 75 owners, 40% agreed to participate in the survey, 53% did not respond, and 6.6% expressed they had no interest in the study. Subsequently, 60% of participants agreed to a follow up one-on-one interview.

### 3.2. Survey Results

The location of the largest number of participants was Queensland (39%), followed by Victoria (25%), New South Wales (18%), Western Australia (11%), and South Australia (7%). There were no respondents from Northern Territory, Tasmania, or the Australian Capital Territory.

**Figure 1:**
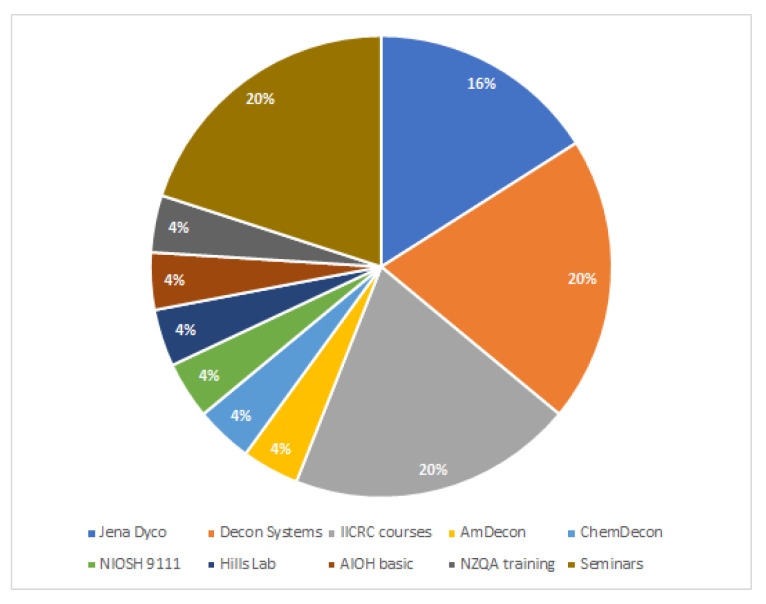
The reported testing and remediation qualifications.

Provided with a free-text box, respondents were asked what testing and remediation qualifications they had. Participants had been trained in equal numbers through Jena Dyco International (now known as TESA Directive), Decon Systems, and the Institute of Inspection Cleaning and Restoration Certification (IICRC) courses and seminars (Figure 1). IICRC is an international organisation that develops standards and offers certified training for cleaning and restoration industries; Jena Dyco International (now trading as TESA Directive) and Decon Systems are both certified Australian training providers for IICRC courses [15,16,17]. The remaining answers were equally divided with single responses between companies and training providers, including Amdecon, Hills Laboratory, the Australian Institute of Occupational Hygienists (AIOH), and New Zealand Qualifications Authority (NZQA).

Forty-eight percent of companies reported ‘less than 10’ queries for methamphetamine remediation services per month and 59% of companies cleaned ‘less than 10’ properties in a month. However, the total number of properties cleaned was bimodal, 23% and 57% reporting ‘less than 10’ or 10–50, respectively, and 30% having cleaned 150–200 properties. This demonstrated a clear divide in the respondents which suggests these enterprises are either large or small, and a few medium-sized enterprises.

### 3.3. Testing Services

**Figure 2:**
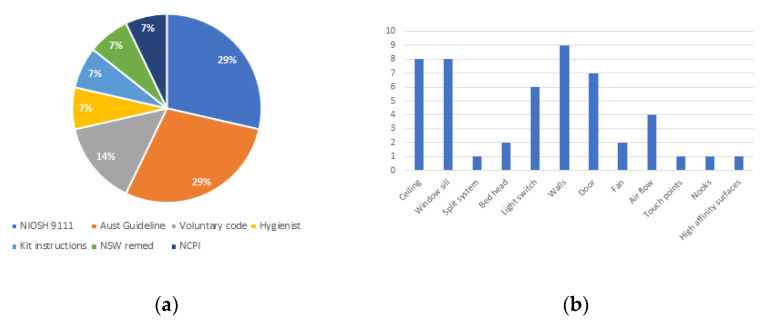
(**a**) Guidelines followed for sampling; (**b**) sampling locations in a contaminated bedroom.

Participants were asked what guidelines they followed for their sampling methods and provided a free-text box for their answer. The most common answers were the National Institute for Occupational Safety and Health (NIOSH) Standard 9111, the Australian guideline 2011, and the Voluntary Code of Practice 2019 (Figure 2a). Other single answers included directions from occupational hygienists, test kit manufacturer instructions, and guidance from the National Contaminated Property Investigations (NCPI).

Companies were asked what services they offered and could select all that applied, which resulted in 76% of participants indicating they offer both testing and remediation while the remainder offered testing (7%) or remediation (17%) exclusively. When asked what type of sampling was used, most companies (44%) used wipe sampling (conducted with gauze wipes and methanol) or 39% used a combination of wipes and presumptive tests (instantaneous presence or absence test). However, there was 17% that used presumptive testing exclusively. For the use of presumptive tests, only 30% stated the tests had been independently validated through a National Association of Testing Authorities (NATA)-accredited laboratory. For those using wipe sampling, 46% used discrete samples, 37% used laboratory composites, with 17% using field composite samples.

When sample wipes were used, those held in separate containers and analysed individually were called discrete wipes. Laboratory composite samples also used individually contained wipes but combined small aliquots of the extracts for analysis. Field composites are wipe samples that were contained, transported, and analysed together [10]. While discrete wipes are the ideal sampling technique, they can easily become a costly screening assessment for the client. Laboratory composite samples can be useful as a cost saving measure and re-assessment of individual samples can be arranged if contamination is present [18]. Field composites, however, are multiple samples held together; therefore, they cannot be re-assessed, and re-sampling of the property needs to occur.

Respondents were asked, with a free-text box, to provide an example of where they would take samples from within a contaminated bedroom. The answers were highly varied and covered a range of surfaces and locations (Figure 2b). Respondents were asked how many photos they would take, which resulted in varied answers. Some respondents stated a number, e.g., two per test or four per room, and others stated a number that would encompass all photos given to the client, e.g., 30–100.

### 3.4. Remediation Services

Seventy percent of respondents stated they prepared a Remediation Action Plan, while the remainder referred to occupational hygienists or the clients preparing the report. Participants were asked whether there were any external factors or stakeholders that would influence surfaces tested and the extent of remediation. This question was focused on contractors and, apart from their own experience, what else had determined the degree of sampling or remediation they would undertake. The AG had the highest response (31%), followed by real estate agents (22%), insurance companies (20%), and the clients (16%), while 11% considered it not applicable.

**Figure 3:**
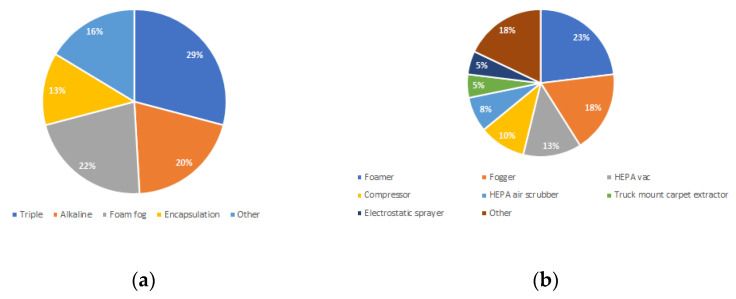
(**a**) Remediation methods used; (**b**) equipment used to remediate methamphetamine contamination.

Participants were asked about the remediation methods used and provided a multiple select option (Figure 3a). The responses predominantly included chemical solutions that were applied directly to the contaminated surface. Triple wash was the preferred treatment, then foam fog and alkaline wash, while encapsulation and other methods had similar response numbers. Fifty percent of participants answered yes to using multiple treatments together. Although companies predominantly used triple wash, foam, and alkaline wash, 20% of companies used the chemicals in conjunction with other treatments, and the most common drying time frame was 24–48 h. Fifty eight percent of respondents had experienced problems with chemical treatments, and it was commonly due to them being incompatible or unsuitable for a particular surface. Participants were asked whether they had a safety data sheet for the chemicals used. Eighty eight percent stated yes; however, the next question asked if they were able to provide the numbers and fewer than half listed them.

A free-text box was provided to respond to a question about the techniques used by respondents to reduce the risk of cross-contamination during remediation. Containment of the areas while cleaning (26%) was the most common answer, followed by separated sample bags (15%), using a HEPA air scrubber (11%), a partial clean of the whole area (11%), changing gloves (11%), and the use of different equipment (11%). The majority of respondents stated that changing gloves was the main cross-contamination prevention measure when sampling, while others mentioned separating samples and covering contaminated surfaces underneath any equipment.

The results from a question about equipment were highly varied (Figure 3b) and many employed multiple pieces of equipment. The two most common were foamers and foggers with 80% of respondents stating they were cleaned after each use. Foggers generate a fine mist of the cleaning solution while foaming machines use compressed air to produce thick bubbles of cleaning solution. Both pieces of equipment are used in other aspects of the cleaning industry [19,20], The remaining 20% of respondents reported cleaning their equipment several times in a day or daily.

With regard to the removal and replacement of electrical appliances from a contaminated property, the results were evenly divided. Participants either stated they would replace all electrical items within a contaminated property, or they would replace items if they were above the recommended 0.5 µg methamphetamine per 100 cm^2^. However, there was one company that stated they would clean all electrical items.

### 3.5. Remediation Outcome

Approximately half of the respondents stated they had, at least once, returned to a property that they had cleaned. When asked why, in most cases it was because a second treatment was required to achieve a concentration below the recommended levels. Fifty eight percent of respondents stated they had remediated a property that had been insufficiently cleaned by another company, with 46% stating this had occurred between one and five occasions. Ten percent stated 5–10 occurrences, 7% had 10–15, 10% stated 15–20 instances, and 7% stated this had occurred over 20 times.

When respondents were asked whether they had consulted other experts for advice, over half of the respondents had contacted an occupational hygienist (55%), many stated another remediator (24%), and other testing companies. Advice from police, insurance companies and Decon Systems was also sought. Again, the number of occasions ranged from high (20+) to very few (1–5), which could be an indication of the size of the company.

### 3.6. One-on-One Interviews

Businesses were contacted once the survey had been completed to allow respondents to elaborate on their survey responses. During this time, conversations often expanded to their experiences of working within the remediation industry. While this was not formally part of the methods of the study, this provided an opportunity to gain further insight based on personal experiences, especially with other industry and associated members. There were 18 respondents that selected yes to participating in an interview after the survey. The following is a combination of the discussions with industry members.

Respondents indicated concerns about issues such as inconsistent sampling results between companies, unrealistically low pricing that could not achieve remediation, and the unethical conduct of some other industry members.

*“A company got a quote from a known dodgy company and contacted me to beat the price. They said the job they wanted done and how much they wanted to pay. I explained that it doesn’t work like that and I’m not interested.”*

Many business owners travel around Australia to secure work in the remediation industry; however, some have been known to not return to reclean a property even if clearance was not successful. Discussions revealed some companies used fog treatment and chose to, subsequently, follow it with water, which had created water damage to properties and, in some cases, did not allow for an accurate validation test. Secondly, some occupational hygienists have also been known to circumvent the Australian Guidelines and provide inadequate information to their clients.

*“There was a recent [validation] report that only provided 3 photos. These clients are paying thousands for a test and [get] very little information.”*

*“[The occupational hygienist] said he forgot to re-test the property, but it was ok, he ‘visually inspected’ it and it was fine.”*

Respondents also expressed concern for the safety of their employees. During one conversation, an owner asked whether their employees were at risk when transporting methamphetamine-contaminated furniture and household items to the local rubbish dump. Upon further questions, it became clear that the company was located in a rural area and, therefore, were having to transport waste a significant distance as hazardous waste removal was not available in the area.

Public perception was also a concern for these business owners. One owner from Queensland stated they were commonly asked by clients if they were required to go through a testing company since Queensland Health Department offers a swab testing service. The owner was obliged to explain there was no legal requirement for them to employ the services of a testing company. They stated it was difficult to demonstrate the challenges of presumptive samples and the benefits of using a testing company when the prices were significantly different.

*“There is a current trend where clients are buying presumptive tests online and testing themselves before they call anyone. This brings about problems with consistency, awareness of where to test and reliability of the test.”*

One respondent mentioned that while rental properties were considered high risk for methamphetamine contamination, they had cleaned approximately 150 homes and the majority of them were owner-occupied properties. Another reported they had positive results after testing for methamphetamine within a property management office.

*“[We] just cleaned a real estate office, where the manager was smoking meth inside the building. So it’s unlikely real estate agents like this will push for property testing in between tenants.”*

## 4. Discussion

The short, ten-minute online survey with a simple user interface that was transferrable to mobile devices was intended to allow business owners to participate without a large time commitment. The combination of quantitative and qualitative questions involved in the survey was aimed at participants’ willingness to share in-depth cleaning and chemical methodology.

### 4.1. Unethical Conduct and Conflict of Interest

The results of the study found that the majority of businesses in the MR industry offered both testing and decontamination services. While it may seem as a natural partnership for the two services, there were ethical implications that must be considered. Business contracts should be based on working for the best interest of the client, both professionally and financially, rather than attracting them through false quality or prices driven by industry competition [21]. Unethical behaviour can be real or perceived; regardless, it can impact how the public perceives the validity of the business. Based on business literature, conflict of interest, deliberate misinformation, or disregard of prior agreements are just a few practices that are recognised as unethical [22]. Therefore, each stage of the decontamination process, the screening and detailed assessments, remediation, and the validation sampling should be offered as separate services by individual companies. Establishing and maintaining a trustworthy reputation is essential for the longevity for any industry [23].

Recommendations have been determined for presumptive testing kits to be independently validated by a NATA accredited laboratory [18]. It is not possible to know the accuracy or precision of a presumptive test without this critical analysis. Therefore, the presumptive test results alone are inadequate to determine the extent of contamination if it was the only method used for detecting methamphetamine. While this method is not strictly unethical, it is not considered best practice.

For results of tests to be analytically valid, a certain number of blanks, controls, and tests for false positives and false negatives with safeguards need to be undertaken [24]. A blank is a test performed on a swab that was known to be uncontaminated. A control is a swab taken to the place of investigation but not exposed. False negatives and positives require a certain number of swabs that were sent for a confirmation analysis to determine if a swab was returning either a negative result but had methamphetamine or a positive result when there was no methamphetamine [25]. These additional tests impose an additional burden in time, resources, and cost. This is an area where unscrupulous companies may cut corners but without these assurance tests the extent of false reporting is not clear.

### 4.2. Lack of Regulation

Currently in Australia, no regulators for the testing and remediation of methamphetamine contamination exist to ensure compliance of the Australian Guidelines. Thus, there is also no simple way for a client to seek a suitable and qualified expert to test or remediate their property. The majority of states and territories were not able to provide a list of reputable testing and remediation companies unless they requested a tender [26]; however, the Department of Health in Western Australia issues lists periodically [27]. The Queensland Health Department offers sampling kits and analytical testing through their NATA accredited laboratory [28]; however, testing knowledge and experience is essential in this instance. Having untrained individuals using test kits could result in inaccurate readings. Other concerns regarding methamphetamine contamination in homes is often addressed by the Environmental Health Officers who are under-resourced and have been impacted by COVID-19 responsibilities [29].

It has been shown that self-regulation can be challenging and that mandated approaches provide structure and compliance [30,31]. For example, to remain compliant with legislative requirements, the food safety industry has adopted auditing strategies that are dependent on the size or franchise status of the company. Auditing can be separated into three segments; self-auditing is based on the individual, internal auditing can be from another sector from within a franchise, and external auditing is from an outside assessor [32]. Both types of internal auditing can provide an outline of issues, while third party auditing would assess the business without any biases or conflicts of interest. Currently, the MR industry is using a self-regulation approach; however, given the public health significance of this issue, external auditing and regulation would be more appropriate.

Many members of the MR industry that were interviewed expressed an interest in, and were willing to advocate for, the establishment of regulatory organisation for the industry. This would provide somewhere for clients to seek advice, accreditation for qualifications and training, and ensure consistency across the industry through audits and other feedback mechanisms. An example of this is the accreditation and licence classes for asbestos removal which are regulated by each state or territory, but are directed by Safe Work Australia [33]. NATA is another certifying authority [34] that could potentially provide accreditation for the use of a standard by licenced testing and remediation companies.

### 4.3. Qualifications and Training

Due to the absence of a regulatory organisation for methamphetamine testing and remediation, there are no accredited MR qualifications available. Therefore, the broad list of MR qualifications and courses provided by participants was likely to be exhaustive. Businesses in the cleaning industry broadened their expertise [35] to meet a market demand in methamphetamine decontamination [15,16].

While not all companies received training from institutions, there were some qualifications—Jena Dyco (now TESA Directive), Decon Systems, and IICRC—that were significantly more common. It has also been acknowledged in the Voluntary Code of Practice (2019), that tertiary education alone is not adequate and that relevant experience within the methamphetamine decontamination industry is paramount [18].

There are training systems that are used to monitor existing companies and to ensure compliance in drug decontamination. In Washington State, Oregon, and Indiana, a mandatory clandestine laboratory cleanup certification has been established to standardise qualifications. These states also have implemented compulsory renewal systems that include refresher courses either annually or every two years [36,37,38].

### 4.4. Protocols and Guidance

This study found a high level of variation in the methamphetamine sampling/testing methods and cleaning techniques used by Australian companies. This can be expected from an industry without standardised methods and the available guidelines are applied on a voluntary basis [11]. However, there were some aspects, such as cross-contamination reduction measures, that appeared to be universally adopted for both sampling and remediation. This is an indication that some methods used for other cleaning services can be successfully adapted to suit methamphetamine decontamination.

The Australian Guidelines and the Voluntary Code of Practice, released in 2011 and 2019, respectively, provided advice and directions for sampling, testing, and remediation of methamphetamine contamination (ACC; AG 2011; Wright 2019). The United States has NIOSH Standards 9111, 9109, and 9106 for the analytical methods (NIOSH 2011a, 2011b, 2011c), and some states provide guidelines for testing and decontamination. A review from Owens [39] published in-depth methods and chemicals from a number of U.S. Government documents and cleaning companies. While it offered great insight, the U.S focus was difficult to obtain comparisons since companies had Proprietary Limited information, and/or they were promoting their own products.

New Zealand’s unified centralised government allows for the incorporation of a standard into legislation to be a more straight-forward process [40]. New Zealand released the Testing and Decontamination of Methamphetamine Contaminated Properties (NZS8510:2017) Standard (Standards New Zealand 2017), and is now moving toward implementing regulations under the Residential Tenancies Amendment Act 2019 [41].

One of the most significant measures that need to be addressed to enable development of a consistent protocol would be for all stakeholders to agree that methamphetamine contamination through personal use, as well as manufacture, is a public health concern. Polarised opinions in this area still remain and this needs to be resolved.

## 5. Conclusions

In Australia and New Zealand, the methamphetamine testing and decontamination industry has grown in response to the awareness of this emerging public health issue. This manuscript provided a foundation for determining common remediation industry practices. These qualitative and quantitative methods can be adopted for other jurisdictions and repeated in their own language. The variation in sampling and cleaning techniques highlights the need for regulation to oversee this industry to ensure consistency within the industry. The establishment of a regulation process would also promote more interdisciplinary collaboration and mitigate unethical practices. Ultimately, the MR industry is comprised of business owners that mainly want to be perceived as an asset and acknowledged that they are working to improve public health. Future research will be aimed at determining the efficacy of the remediation and sampling techniques currently used in the MR industry.

## 6. Limitations

This study was based on a purposive sampling approach to identify MR companies to be contacted. However, participation was voluntary, and this may have introduced some bias and the findings may not be representative of the whole industry. The survey questions were self-driven which allowed the participants to choose what they wanted to answer; however, this resulted in a variation for the interpretation and the number of individual questions answered. The questionnaire design also relied on the attention to detail and honesty of the participants. The available time for business owners to complete the survey was also something to be acknowledged. There were some industry members that stated due to their increased workload from COVID-19 cleaning, they were not able to participate in the study.

## Data Availability

Data is not publicly available, however it can be provided by con-tacting the corresponding author.

## Supplementary Materials

The following are available online at https://www.mdpi.com/article/10.3390/ijerph18178917/s1, Survey S1: methamphetamine remediation survey.
