# Supplementary material for: Overview of Current Practices in the Methamphetamine Testing and Decontamination Industry: An Australian Case Study
Source: Int J Environ Res Public Health. 2021 Aug 25;18(17):8917. doi: 10.3390/ijerph18178917 (PMC8430578; doi:10.3390/ijerph18178917)
Supplement: Supplementary file 1 [file ijerph-18-08917-s001.zip › Survey questions.pdf]

Methamphetamine Remediation Survey

This research project has been approved by the Flinders University Human Research Ethics Committee in South Australia (Project 8634). For queries regarding the ethics approval of this project please contact the Executive Officer of the Committee via telephone on +61 8 8201 3116 or email human.researchethics@flinders.edu.au

Participation for this survey is voluntary. You may choose not to answer any questions and are free to withdraw at any time.

Please read this [Letter of Introduction] for this research

Please read this [Information Sheet] for further details about this online survey

Your company Postcode

____

Do you offer a methamphetamine remediation service to the public?

- ◯ Yes
- ◯ No

Have you been contracted to clean a methamphetamine contaminated property?

- ◯ Yes
- ◯ No

How many queries for the methamphetamine remediation service do you receive per month?

- ◯ I do not receive them on a regular basis
- ◯ Less than 10
- ◯ 10-20
- ◯ 20-30
- ◯ 30+

How many properties would your company clean in a month?

- ◯ I do not clean them on a regular basis
- ◯ Less than 10
- ◯ 10-20
- ◯ 20-30
- ◯ 30+

How many contaminated properties have been cleaned by your company?

- ◯ Less than 10
- ◯ 10-50
- ◯ 50-100
- ◯ 100-150
- ◯ 150-200
- ◯ 200+
- ◯ Not applicable

How many former clandestine laboratories (seized by police) has your company remediated in the last 10 years?

- ◯ Less than 10
- ◯ 10-50
- ◯ 50-100
- ◯ 100-150
- ◯ 150-200
- ◯ 200+
- ◯ Not applicable

What areas does your company service?

- ◯ Central city suburbs
- ◯ Northern suburbs
- ◯ Eastern suburbs
- ◯ Southern suburbs
- ◯ Western suburbs

Are you apart of a franchise?

- ◯ Yes
- ◯ No

How many services does your company offer? (this is the only compulsory question)
Select all that apply

- ☐ Methamphetamine testing
- ☐ Pre-cleaning inspections and testing
- ☐ Post-cleaning inspections and testing
- ☐ Clandestine laboratory remediation
- ☐ Personal use remediation
- ☐ Not applicable

For your testing service, what kind of sampling methods are used?

- ☐ Do It Yourself (DIY) presumptive screening tests
- ☐ Wipe samples (that are analysed in a laboratory)
- ☐ Not applicable

What is the brand/s of the screening tests used?

____

____

____

____

____

Have you independently validated this brand of presumptive test through a NATA accredited laboratory prior to use?

- ◯ Yes
- ◯ No

What kind of analysis is performed on the wipe samples taken?

Select all the apply

- ☐ Discrete (individual) samples
- ☐ Laboratory composite samples
- ☐ Field composite samples

What is the name the laboratory the wipe samples are sent to?

____

What guidance do you use for your sampling methods?

____

____

____

____

____

If there was suspected contamination in a bedroom, where would you sample from?

____

____

____

Do you provide photos to your clients?

- ○ Yes - how many? ____
- ○ No

What techniques are used to reduce the risk of cross contamination of sampling?

____

____

____

____

____

Do you prepare the remediation action plan?

- ○ Yes
- ○ No - who does? ____

Are there external factors that influence the materials and surfaces that you remediate in properties?
Select all that apply

- ☐ Cost limitations from client
- ☐ Instructions from insurance companies
- ☐ Instructions from property managers/owners
- ☐ Guidelines or reports - please specify ____
- ☐ Other____
- ☐ Not applicable

What methods are used by your company to remove methamphetamine contamination?
Select all the apply

- ☐ Triple wash
- ☐ Alkaline wash
- ☐ Foam fog
- ☐ Ozone fog
- ☐ Encapsulation
- ☐ Other____

What time frame do these methods require for airing/drying?

- ○ 1-6 hours
- ○ 6-24 hours
- ○ 24-48 hours
- ○ 48-72 hours
- ○ 72 hours
- ○ Not applicable

Are multiple treatments used together?

- ○ Yes - which ones? ____
- ○ No

How long is the resident required to be away from the property?

- ◯ 1-6 hours
- ◯ 6-24 hours
- ◯ 24-48 hours
- ◯ 48-72 hours
- ◯ 72 hours +
- ◯ Not applicable

Do you have a Safety Data Sheet for the chemicals used for remediation?

- ◯ Yes
- ◯ No

Could you provide the Safety Data Sheet numbers?

____

____

____

____

____

Are these chemicals produced in Australia?

- ◯ Yes
- ◯ No (if not Australia, what country?) ____

Are multiple treatments used together?

- ◯ Yes - which ones? ____
- ◯ No

What techniques are used to reduce the risk of cross contamination?

____

____

____

____

____

Have you found problems with using any of these chemicals?

- ◯ Yes - please specify) ____
- ◯ No

Do you use any specialised equipment?

- ◯ Yes
- ◯ No

Please list equipment used:

____

How frequently do you clean this equipment?

- ◯ After finishing a job
- ◯ Several times a day
- ◯ Daily
- ◯ Weekly
- ◯ Other ____

Have you found problems with using any equipment?

- ◯ Yes - please specify ____
- ◯ No

What is the most common approach to the remediation of electrical items?

- ◯ Remove and replace all
- ◯ Remove and replace some
- ◯ Clean all items

If some items are removed and replaced, please specify which ones.

____

____

____

____

____

If all items are cleaned, please specify what method is used.

____

____

____

____

____

Have you ever had to return to a property that you had cleaned?

- ◯ Yes
- ◯ No

Please describe the reason and the outcome

____

____

____

____

____

Have you been required to remediate a property that was insufficiently cleaned by another company?

- ◯ Yes
- ◯ No

How many occasions has this occurred?

- ○ 1-5
- ○ 5-10
- ○ 10-15
- ○ 15-20
- ○ 20+

Have you ever consulted any of these experts regarding the extent of contamination in a property?

- ☐ Another remediation company
- ☐ Occupational hygienist
- ☐ Building inspector
- ☐ Other ____
- ☐ Not applicable

How many occasions has this occurred?

- ○ 1-5
- ○ 5-10
- ○ 10-15
- ○ 15-20
- ○ 20+

What remediation and/or testing qualifications do you currently hold?

____

____

____

____

____

Would you like to participate in a phone interview to provide more depth to your answers?

◯ Yes

◯ No

For more details about the interview, please read this Information sheet

If you selected Yes for a phone interview, could you please provide some contact details and we will be in touch shortly.

☐ Phone number ____

☐ Preferred time to contact (e.g. Mondays after 1pm) __________________________

Would you like to be sent a copy of the publication or thesis chapter that contains these survey results?

◯ No

◯ Yes (please provide email address) ____
